# Comment on “Screening Value of the I‐Douleur Neuropathique Four Questionnaire for Small Fiber Neuropathy in Patients With Painful Syndromes: Insights From 872 Skin Biopsies”

**DOI:** 10.1111/ene.70564

**Published:** 2026-03-27

**Authors:** Mohammed Anas Mohiuddin, Mohammed Misbah Ul Haq

**Affiliations:** ^1^ Queen Elizabeth Hospital (UHB) Birmingham UK; ^2^ Apollo Hospitals Hyderabad India

**Keywords:** diagnostic screening, DN4 questionnaire, neuropathic pain, skin biopsy, small fiber neuropathy

Dear Editor,

We read with great interest the article by Frachet et al. [1] examining the screening value of the self‐administered DN4 (I‐DN4) questionnaire for small fiber neuropathy (SFN) in a large cohort of 872 patients undergoing skin biopsy. This carefully conducted and methodologically robust study addresses an important and clinically relevant question, given the widespread use of DN4/I‐DN4 in both routine practice and research.

The authors convincingly demonstrated that, despite high sensitivity, I‐DN4 has very poor specificity and essentially no discriminative ability for SFN (AUC ~0.53), even when analyses are restricted to patients with pure SFN and alternative normative IENFD references are applied. These findings challenge the common implicit assumption in clinical pathways that a positive neuropathic pain questionnaire combined with normal nerve conduction studies meaningfully increases the likelihood of SFN [2]. The data presented here strongly suggest that this approach risks substantial overestimation of SFN probability and unnecessary reliance on skin biopsy.

A key strength of this study is the long observation period and consistency of diagnostic procedures, including standardized IENFD quantification by the same investigators over 14 years. The lack of correlation between I‐DN4 scores and both adjusted distal IENFD and the proximo‐distal IENFD ratio further supports the conclusion that symptom‐based neuropathic pain descriptors alone do not reflect underlying small fiber structural damage [2]. This finding is particularly important for clinicians working in pain and rheumatology settings, where DN4‐based screening is frequently used to guide referral decisions.

The observation that only burning pain modestly predicted SFN risk aligns with the pathophysiology of C‐fiber dysfunction, while the association between pinprick hypoesthesia and reduced IENFD reinforces the continued relevance of careful bedside sensory examination. These results support a diagnostic hierarchy in which clinical signs of small fiber dysfunction retain greater specificity than pain descriptors alone.

We also agree with the authors' thoughtful discussion regarding nociplastic pain as a potential explanation for the high prevalence of positive I‐DN4 scores among patients without SFN [3]. This distinction is clinically critical, as misclassifying nociplastic pain as neuropathic may lead to inappropriate investigation and management strategies. In this context, the study highlights a gap between guideline recommendations endorsing DN4/I‐DN4 for neuropathic pain assessment and their limitations when extrapolated to specific disease entities, such as SFN.

Overall, this work provides compelling evidence that I‐DN4 should not be used as a standalone screening tool for SFN. This underscores the need for new disease‐specific bedside tools that integrate quantitative sensory findings and objective biomarkers. Until such tools are available, clinicians should exercise caution in interpreting positive neuropathic pain questionnaires as indicative of small fiber pathology.

## Author Contributions

**Mohammed Anas Mohiuddin:** writing – original draft. **Mohammed Misbah Ul Haq:** conceptualization, writing – review and editing.

## Funding

The authors have nothing to report.

## Ethics Statement

The authors have nothing to report.

## Conflicts of Interest

The authors declare no conflicts of interest.

## Data Availability

The authors have nothing to report.
